# Exploring the Case for a Global Alliance for Medical Diagnostics Initiative

**DOI:** 10.3390/diagnostics7010008

**Published:** 2017-01-26

**Authors:** Melissa L. Mugambi, Kara M. Palamountain, Jim Gallarda, Paul K. Drain

**Affiliations:** 1 Department of Global Health, University of Washington, Seattle, WA 98195, USA; mugambi@uw.edu; 2 Kellogg School of Management, Northwestern University, Evanston, IL 60208, USA; k-palamountain@kellogg.northwestern.edu; 3 Bill and Melinda Gates Foundation, Seattle, WA 98109, USA; jim.gallarda@gatesfoundation.org; 4 International Clinical Research Center, Department of Global Health, University of Washington, Seattle, WA 98195, USA; 5 Division of Infectious Diseases, Department of Medicine, University of Washington, Seattle, WA 98195, USA; 6 Department of Epidemiology, University of Washington, Seattle, WA 98195, USA; 7 Department of Surgery, Massachusetts General Hospital, Boston, MA 02114, USA

**Keywords:** point-of-care diagnostics, global health, low- and middle-income countries, alliance

## Abstract

In recent years, the private and public sectors have increased investments in medical diagnostics for low- and middle-income countries (LMICs). Despite these investments, numerous barriers prevent the adoption of existing diagnostics and discourage the development and introduction of new diagnostics in LMICs. In the late 1990s, the global vaccine community had similar challenges, as vaccine coverage rates stagnated and the introduction of new vaccines was viewed as a distraction to delivering existing vaccines. To address these challenges, the international community came together and formed the Global Alliance for Vaccines Initiative (GAVI). Sixteen years after the formation of GAVI, we see evidence of a healthier global vaccine landscape. We discuss how GAVI’s four guiding principles (product, health systems strengthening, financing and market shaping) might apply to the advancement of medical diagnostics in LMICs. We present arguments for the international community and existing organizations to establish a Global Alliance for Medical Diagnostics Initiative (GAMDI).

## 1. Introduction

In recent years, the private and public sectors have increased investments in the development of diagnostics for low- and middle-income countries (LMICs). Many of these investments have focused on human immunodeficiency virus (HIV), tuberculosis (TB), and malaria because of the concentrated burden of these diseases. These investments have also included support for developing medical diagnostic point-of-care (PoC) testing platforms, due in part to challenges posed by central laboratory systems. While centralized laboratories play an important role in providing medical diagnostic services, they require significant laboratory infrastructure and trained personnel. As patient samples are transported from a network of health facilities in urban and rural settings to centralized laboratories, delays in testing may result in patient loss to follow-up or poor retention in care [1,2]. Decentralized PoC technologies might address the challenges associated with centralized laboratory testing, provided they are easy-to-use, cost effective, provide results within a reasonable time, and yield better patient outcomes [3]. PoC tests continue to supplement, not replace, centralized laboratory testing by bringing services closer to the patient and enable timely availability of test results [4,5].

Historically, there has been poor coordination of efforts between diagnostic test manufacturers and implementation of diagnostic PoC testing in target populations. From the manufacturer’s perspective, there are challenges related to estimating the demand for PoC technologies as well as defining the regulatory and distribution pathways for new diagnostic technologies [6,7,8,9]. From the demand side, many of the barriers relate to the availability of resources to introduce and implement these diagnostics [10,11]. Moreover, it is often unclear which stakeholders lead training and implementation activities. This lack of coordination slows the introduction and uptake of diagnostic technologies and limits the degree to which improved patient outcomes are realized.

These challenges are not unique to the global medical diagnostics community. The global vaccine community experienced similar challenges when introducing and implementing vaccines in LMICs. In the late 1990s, global immunization rates had become stagnant. By 2000, children in developed-word countries were receiving an average 11–12 vaccines while children in poor countries received half that number [12]. Nearly 30 million children in developing countries were not fully immunized. Newer, more expensive vaccines routinely given to infants in the rich world, such as the hepatitis B (HepB) and *Haemophilus influenzae* type b (Hib), were reaching few of the world’s poorest children [12]. As a result of these challenges, the international community organized the Global Alliance for Vaccines Initiative (GAVI) to speed the introduction of children’s vaccines in LMICs [13]. As of 2015, GAVI had successfully vaccinated half a billion children and saved seven million lives [14].

The global medical diagnostics community does not have a similar global alliance to coordinate the development, introduction, and utilization of medical diagnostics. Instead, numerous agencies and partnerships have engaged in the diagnostics market and supporting country implementation (Table 1), but each agency and project has individual competencies, objectives, funding, and governance models. In this paper, we explore the GAVI model and how the global medical diagnostics community could apply similar principles to better achieve impact.

## 2. Overview of Global Alliance for Vaccines Initiative (GAVI)’s Strategic Framework

Prior to the formation of GAVI, many organizations and individuals debated how to improve coverage rates across existing vaccination programs, and also how to increase the number of new vaccines introduced in LMICs [13]. As a result of four critical meetings, GAVI was created in 2000 to address market barriers and improve vaccination coverage in over 70 LMICs. To ensure that children have equal access to vaccines, GAVI applies the following four principles to create a healthy vaccine market [15]:

1. The vaccine goal: Accelerate the uptake and use of underused and new vaccines by strengthening country decision-making and introduction.
2. The health systems goal: Contribute to strengthening the capacity of integrated health systems to deliver immunizations.
3. The financing goal: Increase the predictability of global financing and improve the sustainability of national financing for immunization.
4. The market shaping goal: Ensure development of appropriate and affordable vaccines for developing countries.

Although this strategic framework is strictly focused on sustainable implementation of vaccine products in developing countries, it is important to consider its applicability to global health interventions such as diagnostics. These are considered below.

## 3. Accelerating the Uptake and Use of Products

One of GAVI’s goals specifically focuses on vaccine product availability [15]. Broad consensus on which vaccines are most essential to human health dates back to 1974, when the World Health Organization established the Expanded Programme on Immunization (EPI) as part of a wider initiative to improve access to primary health care services [16]. Built on a successful smallpox eradication logistics network, the EPI’s goal was to improve access to vaccines for six recommended target diseases that were selected based on disease burden and vaccine availability. The EPI program encouraged countries to integrate certain vaccines into their national immunization programs [17,18]. Over the life of the program, additional vaccines were added to routine immunization schedules—namely hepatitis B and yellow fever. GAVI was subsequently established to bolster EPI program efforts and to improve immunization coverage. Since then, GAVI has focused on the planning, adoption, and implementation of vaccines by helping countries decide how and when vaccines should be introduced, how they might be sustainably financed, and how a sufficient supply of vaccines and vaccine-related products can be ensured [15].

Unlike vaccines or medicines, a globally endorsed essential list for medical diagnostics has been proposed, but not established [19]. In some cases, countries have an existing test menu or product guide that specifies the types of testing methods and corresponding technologies that should be conducted at each facility level. In other cases, countries do not explicitly define essential medical diagnostics, leaving gaps in quality and accessibility of critical diagnostic tests [20]. Therefore, a globally coordinated effort to identify essential medical diagnostics will not only be useful in determining what existing diagnostic products are presently underused, but also which products innovators might choose to develop because they either do not exist or have not been designed for use in LMICs. When it comes to procuring specific brands of diagnostics on the essentials list, GAMDI could utilize a similar approach to GAVI, where the specific product is either pre-qualified by the World Health Organization (WHO) or a stringent regulatory authority (e.g., United States Food & Drug Administration). This would help ensure the quality of the diagnostic and a well-controlled manufacturing process. Individual countries often have their own registration and approval process for specific diagnostic tests as well. GAMDI could help provide transparency around the path to regulatory approval in each country and perhaps fund trials to measure product performance in countries with significant socio-demographic differences.

## 4. Strengthening the Capacity of Integrated Health Systems

Initially, GAVI’s health system strengthening efforts were focused on the vaccine products. However, wider systemic barriers to the uptake of these vaccines prompted GAVI, as a public–private coalition of existing stakeholders, to adopt a broader strategy addressing country challenges to the health workforce, equipment, infrastructure, and management. As access to primary health care services improved, GAVI focused on improving vaccine delivery to hard-to-reach areas by addressing logistics, supply chain, and health information systems [15].

Health system strengthening has also become an important agenda item for donor agencies involved in funding the procurement of PoC diagnostic technologies. For example, the Global Fund to Fight AIDS, TB and Malaria (Global Fund) adopted a cross-cutting strategy that enables countries to apply for funding to support interventions. These interventions largely focus on health workforce, delivery systems, supply chain, procurement, health financing, leadership, and governance, all of which can generate spillover benefits for other diagnostic interventions [21]. Conversely, efforts to strengthen health systems directly linked to medical diagnostics have been in a nascent state [22]. Medical diagnostics can require complex post-implementation support for system installation, training, service and maintenance, distribution and storage, quality control and assurance, data connectivity platforms to inform patient management, and monitoring and surveillance activities [22].

There has been little clarity as to who is primarily responsible for the post-implementation support for medical diagnostics. Product developers, implementing partners, national governments, and international institutions play different roles with their own competencies, goals, funding and governance models. In higher-income countries, the manufacturer itself takes a lead role in working with heads of hospitals and clinicians in those hospitals, along with laboratories to address such post-implementation support needs, many of which are required by regulatory agencies. This operational model can be difficult in developing countries where engaging with key stakeholders and navigating the healthcare and regulatory systems can be more challenging. Organizations such as the African Society for Laboratory Medicine and Asia Pacific Laboratory Accreditation Cooperation could play a pivotal role as their reach extends beyond HIV and TB and more broadly across all diseases served through a country’s healthcare system. However, ongoing efforts to strengthen laboratory systems and to promote stronger leadership and governance structures are in their early stages [10].

## 5. Increasing the Predictability of Global Financing and Improving the Sustainability of National Financing

Predictable, long-term financial support for vaccines is another goal of the GAVI model. Indeed, GAVI chose to emphasize financial sustainability from its inception [23]. Achieving this financial support not only gives countries the confidence to introduce new vaccines, but by providing visibility of future demand, GAVI enables manufacturers to better plan their production of vaccines at more affordable prices. Such financial support and demand transparency also serve as incentives for new manufacturers to enter these emerging markets. GAVI is a public–private partnership with a unique governance model that aligns interests among formerly non-coordinated players. GAVI funding comes from governments, corporations, foundations, private individuals and, importantly, capital markets. GAVI has secured over US $20 billion in financing, which will fund vaccine activities through 2020 [15]. To achieve this long-term financing, GAVI deployed a novel financing mechanism through the International Finance Facility for Immunization (IFFIm). This model creates readily available cash resources by using government pledges to back the issuance of bonds on the capital markets. To address sustainability, the GAVI governance model also asks lower-income countries to increase their own investment in immunization programs with a detailed Financial Sustainability Plan, which describes how countries would transition from GAVI funds to other sources. While co-financing from lower-income countries is small (US $113M in 2015) relative to larger donors (US $1.6B in 2015), lower-income countries have increased their own investment per child from US $3.80 per child in 2010 to over US $6.30 per child in 2015 [15]. Moreover, four countries are set to transition completely out of GAVI in 2016, with a long-term goal of transitioning out 20 countries by 2020. With GAVI’s initial infusion of financial resources for vaccines and the subsequent benefits, the case for vaccine investment becomes clearer to lower-income countries, as vaccine coverage increases, vaccine prices decrease, and the under-five child mortality rates decrease.

A number of organizations have funded diagnostics, oftentimes without considering long-term sustainability or overlapping efforts. The Bill & Melinda Gates Foundation (BMGF) has provided funds for medical diagnostic product development across multiple diseases including HIV, TB, malaria, neglected tropical diseases, Ebola, and enteric and diarrheal diseases. The BMGF has also funded the introduction and implementation of diagnostic products for human African trypanosomiasis and HIV self-testing. UNITAID has funded the introduction of new tests, including the introduction of early infant diagnostic testing systems for HIV as well as for case detection for TB. The Global Fund and the United States President’s Emergency Plan for AIDS Relief (PEPFAR) have provided significant infusion of funding for HIV antibody tests, CD4 testing systems, and most recently HIV viral load nucleic acid test (NAT) systems. However, there is little coordination among these varying funding agencies, and the multiplicity of grant application processes can seem daunting to non-governmental organizations (NGOs), local governments, and product developers. Until recently, there has been little call for innovative financing such as GAVI’s IFFIm that leverages capital markets popular with institutional and individual investors and that provides prompt access of funds to GAVI programs. The Stop TB partnership has recently proposed a social impact fund called Venture Lab to pool and blend private and public capital to accelerate development and scale of TB diagnostic tools through sustainable business partnerships between developers and country governments [24].

## 6. Ensuring Appropriate and Affordable Products for Developing Countries

One of the primary goals of GAVI is to ensure that vaccine markets work better for lower-income countries. To achieve this goal, GAVI provides a coordinated, aggregated forecasting mechanism that allows manufacturers to plan production based on known demand, donors to maximize their investments and, most importantly, developing countries to buy suitable vaccines at prices they can afford and to eventually transition out of GAVI support.

Since 2000, GAVI has helped to increase demand certainty by aggregating vaccine demand in over 70 countries for 10 vaccines focused across six leading vaccine-preventable diseases [15]. UNICEF manages this demand forecast through a pooled procurement system that GAVI then pairs with long-term donor commitments to increase forecast certainty. The demand certainty then enables manufacturers to plan production more effectively—reducing the risk of supply shortages and allowing GAVI to obtain lower prices. This has resulted in a reduction in vaccine prices, namely the pentavalent rotavirus and pneumococcal vaccines, which have decreased in price by 43% since 2010 [15].

GAVI’s efforts to foster healthy vaccine markets have led to increased competition and a more diversified manufacturing base. In 2001, there were only five GAVI vaccine suppliers in five countries; by the end of 2015, 16 manufacturers in 11 countries were producing prequalified vaccines suited to the needs of GAVI-supported countries.

Similar demand forecasting initiatives have emerged in medical diagnostics, although typically focused on individual diseases. One example is the Clinton Health Access Initiative Procurement Consortium, which provides forecasts to suppliers for 70 countries in return for discounted prices for HIV laboratory consumables (e.g., the disposable elements required per test). However, in addition to the number of testing consumables that are projected, forecasts for test-associated diagnostic instruments are also required. For central laboratory-based instrument systems, the estimate is relatively straightforward since typically only few laboratories in a country can accommodate such sophisticated equipment. For more decentralized PoC platforms, however, a larger number of instruments is required. Regardless, whether the instrument is designed for larger centralized labs or placement at the clinical point of care, manufacturers often design machines for several tests rather than a single disease application. Thus, disease-specific forecast initiatives make it difficult to estimate the number of instruments required to keep up with the daily volume of tests in health facilities with patients needing tests for multiple diseases. The WHO Unified Xpert MTB/RIF Forecasting Initiative provides aggregated forecasts for the GeneXpert instrument and associated Xpert MTB/RIF TB test cartridges. This group has recognized that new multi-disease PoC platforms from other manufacturers are on the horizon and that collective planning with other initiatives is needed [25].

## 7. The Case for a Global Alliance for Medical Diagnostics Initiative (GAMDI)

Since many of the issues GAVI faced are pertinent to medical diagnostics, it is reasonable to explore whether GAVI’s governance model is appropriate to medical diagnostics in global health. To apply the GAVI model to diagnostics, the diagnostics community needs a more thorough understanding of the current roles of existing players. First, there needs to be a comprehensive description of existing key stakeholders that work across the four guiding principles of GAVI (product, health system strengthening, financing and market shaping). As part of their academic research, the authors of this paper are willing to take the lead in interviewing individual organizations in Table 1. Once this mapping is complete, broad stakeholder meetings should be convened to identify areas of overlap and opportunities to increase the uptake of existing and new diagnostics through a GAVI-like model for medical diagnostics. A primary issue will be the development of a global essential diagnostics list. Developing the investment case for diagnostics on this essential list will require a clear link between the diagnostic tools and the health gap addressed such that the return on the investment in health is measurable.

If an essential list for diagnostics is developed, a GAVI-like model could then focus on improving access to these essential diagnostics. Improved transparency around regulatory systems, data connectivity systems, transportation networks, medical waste management, and consistencies in power supply are all fundamental infrastructural needs that are common to various diagnostic technologies. Addressing these needs could make a substantial difference in how quickly and effectively diagnostics are scaled. Furthermore, there are many partners, spanning research that could contribute to this effort, including those listed in Table 1. Having one organization such as GAMDI—a GAVI for diagnostics—coordinating the supply and demand of diagnostic systems will maximize the price reductions for participating countries.

Sustainability in diagnostics requires predictable, long-term financing. Funding for essential diagnostics should not only come from donors but also the countries themselves (such local investment will be evidence that these diagnostics are in fact essential). The funding also needs to be assured—initially, GAVI used vaccine bonds and the same could be applied to diagnostics. However, sustainable financing could be assured with commensurate country commitment to develop durable financial sustainability plans. Using government pledges to back the issuance of diagnostic bonds on the capital markets would provide predictable cash resources for GAMDI. These funds could then be used to introduce and implement diagnostics, strengthen the health systems surrounding these essential diagnostics, and create incentives for the development of new diagnostics.

Future investments in diagnostics require novel ways to reduce barriers and increase incentives for developing and implementing diagnostics. Applying the principles of the GAVI model, we can create GAMDI, which may remove barriers in the diagnostics market with the end goal of improving access to diagnostics and improving the health of people worldwide.

## Figures and Tables

**Table 1:** Partners in the Global Medical Diagnostic Community.

#	Agency	#	Agency
1	Ministries of Finance & Health from Global Alliance for Vaccines Initiative (GAVI)-eligible countries	25	International Diabetes Federation (IDF)
2	Accordia Global Health Foundation	26	International Diagnostics Centre (IDC)/London School of Hygiene and Tropical Medicine (LSHTM)
3	African Society for Laboratory Medicine (ASLM)	27	International Training and Education Center for Health (I-TECH)/University of Washington
4	Asia Pacific Laboratory Accreditation Cooperation	28	In Vitro Diagnostic (IVD) manufacturers & distributors
5	American Association for the Advancement of Science (AAAS)	29	Joint United Nations Programme on HIV/AIDS (UNAIDS)
6	American Society for Clinical Pathology (ASCP)	30	Kaiser Family Foundation (KFF) USA Global Health Policy
7	American Society for Microbiology (ASM)	31	Malaria no More
8	American Society of Tropical Medicine and Hygiene (ASTMH)	32	Medicins Sans Frontieres (MSF)
9	American Thoracic Society (ATS)	33	Partnership for Supply Chain Management (SCMS)
10	Asia Pacific Laboratory Accreditation Cooperative	34	PATH
11	Bill and Melinda Gates Foundation (BMGF)	35	Research!America Global Health R&D Advocacy
12	Center for Strategic and International Studies (CSIS) Global Health Policy Center	36	Stop TB Partnership
13	Clinton Health Access Initiative (CHAI)	37	The Earth Institute
14	Consortium of Universities for Global Health (CUGH)	38	The Global Fund to Fight AIDS, Tuberculosis, and Malaria (The Global Fund)
15	CRDF Global	39	The Global Health Network
16	Foundation for Innovative New Diagnostics (FIND)	40	UN Foundation (UNF)
17	Foundation for NIH (FNIH)	41	UNITAID
18	GBCHealth	42	United States Agency for International Development (USAID)
19	Global Alliance for Chronic Diseases (GACD)	43	United States Center for Disease Control (CDC)
20	Global Health Council	44	United States National Institutes of Health (NIH) e.g., National Institute of Biomedical Imaging and Bioengineering Point-of-Care Technology Research Network
21	Global Health Innovative Technology Fund (GHIT)	45	United States President's Emergency Plan for AIDS Relief (PEPFAR)
22	Global Health Security Agenda (GHSA)	46	Wellcome Trust
23	Global Health Technologies Coalition (GHTC)	47	World Bank
24	Infectious Diseases Society of America (IDSA)	48	World Health Organization (WHO)
